# Aromadendrin Inhibits T Cell Activation via Regulation of Calcium Influx and NFAT Activity

**DOI:** 10.3390/molecules25194590

**Published:** 2020-10-08

**Authors:** Hyun-Su Lee, Gil-Saeng Jeong

**Affiliations:** College of Pharmacy, Keimyung University, 1095 Dalgubeol-daero, Daegu 42601, Korea; hyunsu.lee@kmu.ac.kr

**Keywords:** T cells, IL-2, calcium, NFAT, MAPK

## Abstract

The objective of this study was to assess the inhibitory effect of the flavonoid aromadendrin on T cell activity to identify a non-cytotoxic immunosuppressive reagent. Conventional and qualitative PCR, MTT assays, flow cytometry and Western blotting were used to evaluate the effect of aromadendrin on the activity, cell viability and confluency, and proximal signal transduction of activated T cells. Aromadendrin effectively regulated IL-2 and IFNγ production in vitro from activated Jurkat T cells without cytotoxicity. Pre-treatment with aromadendrin also suppressed the expression levels of surface molecules CD69, CD25, and CD40L. Reduced calcium (Ca^2+^) influx in activated T cells pre-treated with aromadendrin was observed. Western blotting revealed that aromadendrin blocked the dephosphorylation of nuclear factor of activated T (NFAT) cells and its nuclear translocation. Involvement of the NFκB and MAPK pathways in the inhibitory effect of aromadendrin was also demonstrated. Results obtained demonstrated the suppressive effect of aromadendrin on T cell activation by Ca^2+^ influx regulation through NFAT activity suppression of the activated T cells.

## 1. Introduction

T cell activation plays a central role in adaptive immune responses of other immune cells [1], including helping B cells to produce antibodies [2]. Several therapeutic strategies for T cell mediated diseases have demonstrated that regulation of excessive T cell activation during pathogenesis by immunosuppressive reagents is key; however, research on non-cytotoxic and effective bioactive compounds from natural products that possess a regulatory effect on T cell activation is limited. To enhance effective immune responses, T cells transduce intracellular signals from their surfaces to the nucleus [3] using intracellular messenger molecules such as calcium (Ca^2+^) ions that regulate the “switch on” and “switch off” function of T cells [4]. The concentration of these cytosolic calcium ions is regulated by the calcium permeable channels and transporters expressed on the T cell surfaces to maintain calcium signaling. Calcium influx is, however, stimulated by signal mediation with T-cell receptor (TCR) [5]. An antigenic signal from engagement with antigen presenting cells (APCs) leads to a cascade that also promotes calcium influx [6]. The cascade including dephosphorylation of Lck, phosphorylation of ZAP70 and phospholipase Cγ1 (PLCγ1) produces Inositol-1,4,5-triphosphate (IP3) by PLCγ1 that binds to the receptor expressed on the ER (endoplasmic reticulum) membrane, and promotes calcium release into the cytosol [6,7]. Calcium ion influxes cause dephosphorylation of nuclear factor of activated T (NFAT) cells and translocation into nucleus, and T cell-related gene induction by the NFκB pathway [8,9,10]. Though controlling the calcium level in cytosol is pivotal in T cell activation, research on the suppressive effect on calcium signaling in T cell by bioactive compounds from natural products is limited.

**Figure 1:**
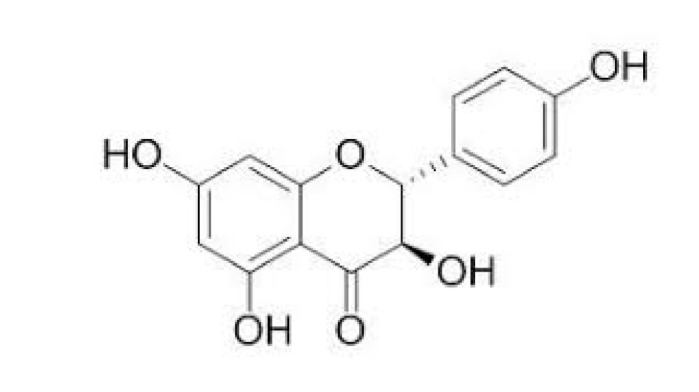
The chemical structure of aromadendrin.

Aromadendrin (Figure 1) is a flavonol widely found in a variety of plants including *Chionanthus retusus*, *Pinus sibirica*, and *Afzelia bella* [11,12,13]. It possesses several bioactivities including anti-tumor, radical scavenging, anti-inflammatory, and anti-diabetic activities [11,14,15,16]. The novel inhibitory effect of aromadendrin on cardiac hypertrophy, via suppression of the NFAT and MAPK pathway, in rat neonatal ventricular cardiomyocytes (RNVMs) has been reported [17]. Despite research on the discovery of the pharmacological properties of aromadendrin, little is known of its role in controlling activity of activated T cells. The present study evaluated the suppressive effect of aromadendrin on T cell activation via the downregulation of Ca^2+^ influx and NFAT activity in activated T cells.

## 2. Result

### 2.1. Aromadendrin Does Not Affect T Cell Viability

**Figure 2:**
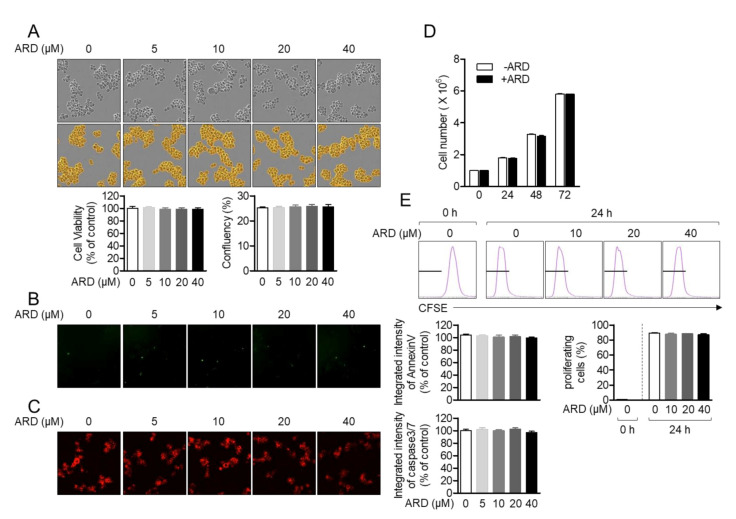
Aromadendrin does not affect T cell viability. (**A**) Cell viability, confluency, (**B**) the expressed AnnexinV, and (**C**) caspase3/7 of Jurkat T cells incubated with 40 μM of aromadendrin for 24 h were determined by MTT assay (**A**) and the IncuCyte imaging system. (**D**) The growth rate of Jurkat in the presence of 40 μM aromadendrin was determined by counting the cell number up to 72 h. (**E**) The percentage of proliferating cells in the presence of aromadendrin was obtained by CFSE (carboxyfluorescein succinimidyl ester) proliferation assay. The mean value ± SEM (stand error of the mean) are presented in the bar graph.

Toxicity of aromadendrin on Jurkat T cells was first estimated. Cellular viability and confluency of Jurkat T cells in the presence of aromadendrin were confirmed by MTT assay and the IncuCyte cell imaging system. Figure 2A revealed that treatment with up to 40 μM aromadendrin did not show cytotoxicity nor confluency change in Jurkat T cells. To confirm whether aromadendrin induced the apoptosis pathway, the expression of AnnexinV and caspase3/7 was observed by the IncuCyte imaging system. Integrated intensities of AnnexinV and caspase3/7 from cells incubated with up to 40 μM aromadendrin were not significantly changed. The 40 μM of aromadendrin did not affect the growth rate of Jurkat T cells up to 72 h (Figure 2D). The result from the CFSE (carboxyfluorescein succinimidyl ester)-proliferation assay also confirmed that aromadendrin is not suppressive to cell proliferation (Figure 2E). These data suggest that aromadendrin does not lead to cytotoxicity including apoptosis in Jurkat T cells.

### 2.2. Aromadendrin Suppresses Pro-Inflammatory Cytokines from Activated T Cells

**Figure 3:**
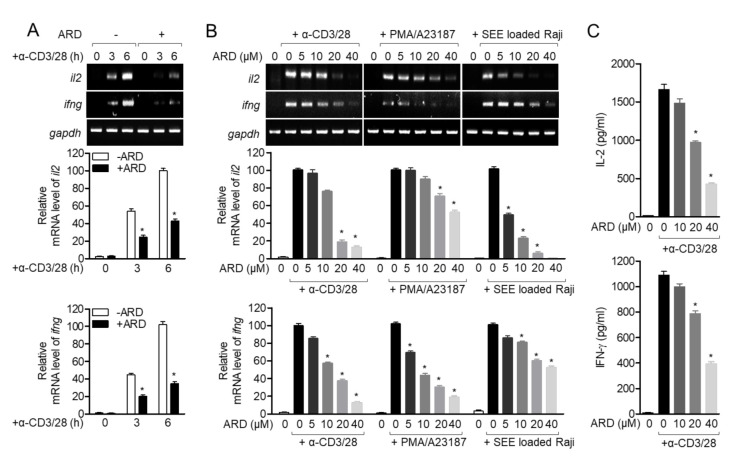
Aromadendrin suppresses cytokine production from activated T cells. (**A**) Jurkat T cells pre-treated with 40 μM aromadendrin for 1 h were stimulated with anti-CD3/CD28 antibodies for the indicated time, and mRNA levels of il2 and ifng were determined by conventional and real-time PCR. (**B**) Jurkat T cells were pre-treated with 40 μM aromadendrin for 1 h and stimulated with anti-CD3/CD28 antibodies, PMA/A23187, or SEE-loaded Raji B cells to detect the mRNA levels of il2 and ifng by conventional and real-time PCR. (**C**) Production of IL-2 and IFNγ from activated Jurkat T cells pre-treated with 40 μM aromadendrin was detected by ELISA. The mean value ± SEM are presented in the bar graph. * *p* < 0.05, versus the cells activated by anti-CD3/CD28 antibodies.

To assess whether pre-treatment with aromadendrin of Jurkat T cells exerted an inhibitory effect on T cell activity, mRNA levels of il2 and ifng were measured by conventional and quantitative PCR in activated T cells using anti-CD3/CD28 antibodies. As shown in Figure 3A, Jurkat T cells pre-treated with aromadendrin exhibited inhibition in *il2* and ifng expression. A dose-dependent experiment was also performed to investigate whether the regulatory effect of aromadendrin was dependent on the stimulating method of T cells. Figure 3B shows that the pre-treatment with aromadendrin possesses suppressive effect on the expression of *il2* and *ifng* from activated T cells by anti-CD3/CD28 antibodies, PMA/A23187, or SEE-pulsed Raji B cells. It was confirmed whether pre-treatment with aromadendrin affected downregulation in IL-2 and IFNγ proteins from activated T cells. ELISA data revealed that aromadendrin regulated IL-2 and IFNγ production from activated T cells (Figure 3C). These results demonstrated that aromadendrin effectively inhibited activated T cell activity from generating pro-inflammatory cytokines.

### 2.3. Aromadendrin Reduces the Expression of Surface Molecules in Activated T Cells

**Figure 4:**
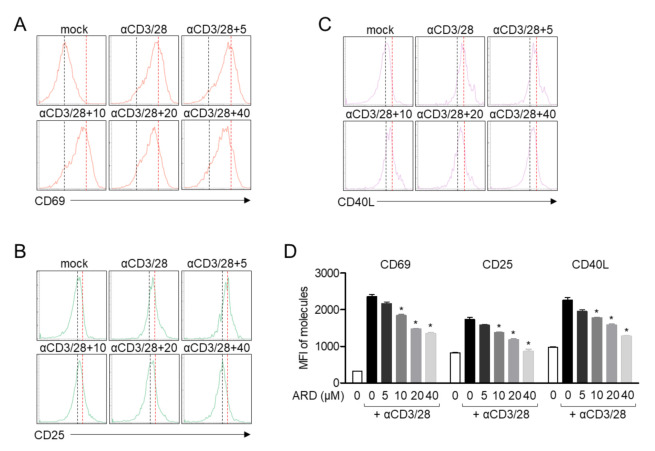
Aromadendrin reduces the expression of surface molecules in activated T cells. Jurkat T cells, pre-treated with 40 μM of aromadendrin for 1 h, were stimulated with anti-CD3/CD28 antibodies for 16 h (**A**) or 24 h (**B**,**C**). Collected cells were stained with anti-CD69, anti-CD40L, and anti-CD25 antibodies conjugated with APC (allophycocyanin) and the expression was analyzed by flow cytometry. (**D**) The mean value ± SEM of CD69, CD25 and CD40L are presented in the bar graph. * *p* < 0.05, versus the cells activated by anti-CD3/CD28 antibodies.

It has been reported that the expression patterns of surface molecules are dramatically changed according to their functions [18]. In order to estimate whether pre-treatment with aromadendrin had regulatory effects on the expression of surface molecules in activated T cells, the expressions of CD69, CD25, and CD40L were assessed by flow cytometry. CD69, a marker of early T cell activation, was suppressed by pre-treatment with aromadendrin (Figure 4A). The expression of CD25, a receptor of IL-2, was also reduced in Jurkat T cells pre-treated with aromadendrin (Figure 4B). Figure 4C shows that CD40L, critical as a co-stimulatory molecule in activated T cells, is significantly downregulated in an aromadendrin concentration-dependent manner. These data suggest that aromadendrin abrogates overall activity of T cells including surface molecule expression.

### 2.4. Aromadendrin Inhibits Ca^2+^ Mobilization in Activated T Cells

**Figure 5:**
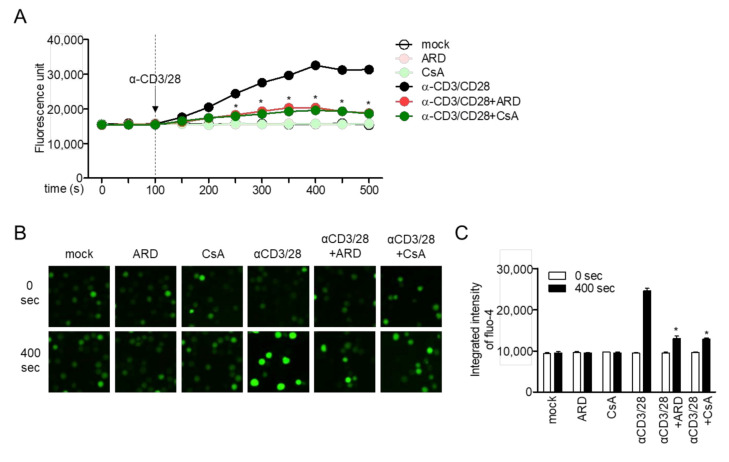
Aromadendrin inhibits Ca^2+^ mobilization in activated T cells. Stained Jurkat T cells with 4 μM fluo-4 were pre-treated with 40 μM aromadendrin or 2.5 μM CsA for 1 h, and soluble anti-CD3/CD28 antibodies were added to the cells for stimulation at 100 s. (**A**) Increased Ca^2+^ influx was assessed by an Immunoabsorbance reader and (**B**) IncuCyte. (**C**) The mean value ± SEM of integrated intensity of fluo-4 are presented in the bar graph. * *p* < 0.05, versus the cells activated by anti-CD3/CD28 antibodies.

Immediate influx of calcium into cytosol is a critical event in TCR-mediated stimulation for signal transduction from membrane to the nucleus. To explore whether aromadendrin affected calcium mobilization in the activated condition, enhanced fluorescence was determined in activated T cells pre-stained with fluo-4 staining dye exclusively binding to calcium. The effect of activated T cells pre-treated with cyclosporin A (CsA), a calcineurin inhibitor, on fluo-4 fluorescence was also determined. Treatment with aromadendrin did not show changes in calcium influx on resting T cells but significantly reduced fluorescence in activated T cells pre-treated with aromadendrin (Figure 5A). Comparable changes in calcium influx were observed in activated T cells pre-treated with CsA (Figure 5A). Microscopic images were captured and examined to confirm whether aromadendrin had an inhibitory effect on cytosolic calcium influx. Figure 5B shows that pre-treatment with aromadendrin downregulates calcium influx in the presence of TCR-mediated stimulation. These data suggest that TCR-mediated stimulation promotes cytosolic calcium influx in a calcineurin-independent manner, whereas aromadendrin blocks T cell activation via cytosolic calcium influx.

### 2.5. Pre-Treatment with Aromadendrin Controls NFAT Activity in Activated T Cells

**Figure 6:**
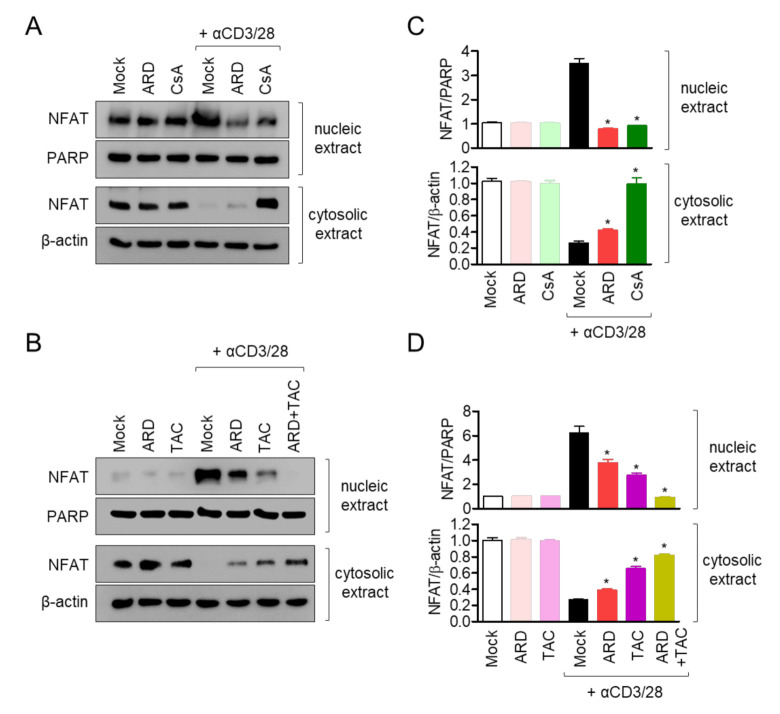
Pre-treatment with aromadendrin controls nuclear factor of activated T (NFAT) cells activity in activated T cells. (**A**) Jurkat T cells pre-treated with 40 μM aromadendrin or 1 μM CsA for 1 h, and stimulated with anti-CD3/CD28 antibodies for 1 h. (**B**) Jurkat T cells pre-treated with 40 μM aromadendrin and/or 2.5 ng/mL tacrolimus for 1 h, and stimulated with anti-CD3/CD28 antibodies for 1 h. Nucleic acid extracts were separated from whole lysates by a NE-PER^TM^ kit and the localization of NFAT was determined by Western blotting. (**C**,**D**) Nucleic acid extract was normalized with the expression of PARP cytosolic extract that was normalized with the expression of β-actin. The mean value ± SEM are presented in the bar graph. * *p* < 0.05, versus the cells activated by anti-CD3/CD28 antibodies.

NFAT has been reported as one of the pivotal pathways in *il2* gene transcription in T cells which is translocated by calcineurin activity. To verify whether pre-treatment with aromadendrin affected NFAT translocation into nucleus, the expression of NFAT was detected by Western blot analysis in nucleic and cytosolic extracts. Figure 6A shows that less cytosolic NFAT is transferred into nucleus in activated T cells pre-treated with aromadendrin compared to activated T cells pre-treated with CsA. To confirm whether pre-treatment with aromadendrin blocks NFAT translocation in activated T cells, we used a different calcineurin inhibitor, tacrolimus. Pre-treatment with tacrolimus also showed a suppressive effect on NFAT translocation. Interestingly, co-treatment with aromadendrin and tacrolimus exhibited a complete block of NFAT translocation in activated T cells (Figure 6B). These data demonstrate that aromadendrin regulates NFAT activity in activated T cells.

### 2.6. Aromadendrin Suppresses p65 Translocation and MAPK but Not Proximal Pathway

**Figure 7:**
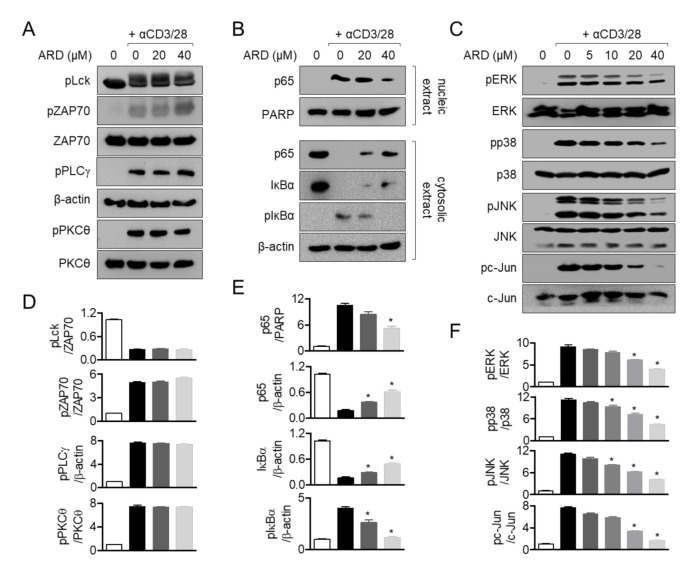
Aromadendrin suppresses p65 translocation and the MAPK but not proximal pathway. (**A**) The phosphorylated protein levels of activated Jurkat T cells pre-treated for 10 min with 20 or 40 μM of aromadendrin were assessed by Western blotting. (**B**) p65 translocation into nucleus was detected by Western blotting using activated Jurkat T cells pre-treated for 1 h with 20 or 40 μM of aromadendrin. (**C**) The phosphorylated protein levels of activated Jurkat T cells pre-treated for 30 min with 20 or 40 μM of aromadendrin were assessed by Western blotting. (**D**–**F**) The mean values ± SEM of each protein indicated are presented in the bar graph. * *p* < 0.05, versus the cells activated by anti-CD3/CD28 antibodies.

Proximal signaling transduction in response to TCR-mediated stimulation is essential for early phase T cell activation. To evaluate whether aromadendrin affected proximal signaling pathway, phosphorylated levels of Lck, ZAP70, PLCγ1, and PKCθ from activated T cells were measured by Western blotting using anti-CD3/CD28 antibodies. Figure 7A reveals that pre-treatment with aromadendrin is not associated with the inhibition of proximal signal pathways. As MAPK, through NFκB, is also important for T cell activation, we elucidated whether pre-treatment with aromadendrin was involved in the downregulation of the MAPK pathway and p65 translocation in activated T cells. Translocation of p65 was partially blocked by pre-treatment with aromadendrin in activated T cells. Besides, phosphorylation and degradation of IκBα were slightly abrogated (Figure 7B). Phosphorylation levels of MAPK pathway including ERK, p38, JNK, and c-Jun were suppressed by pre-treatment with aromadendrin in activated T cells (Figure 7C). These data suggest that aromadendrin regulates the MAPK pathway through inhibition of p65 activity but not proximal signals in activated T cells.

### 2.7. Aromadendrin Induces Nrf2 Pathway and Acts as an Antioxidant in Activated T Cells

**Figure 8:**
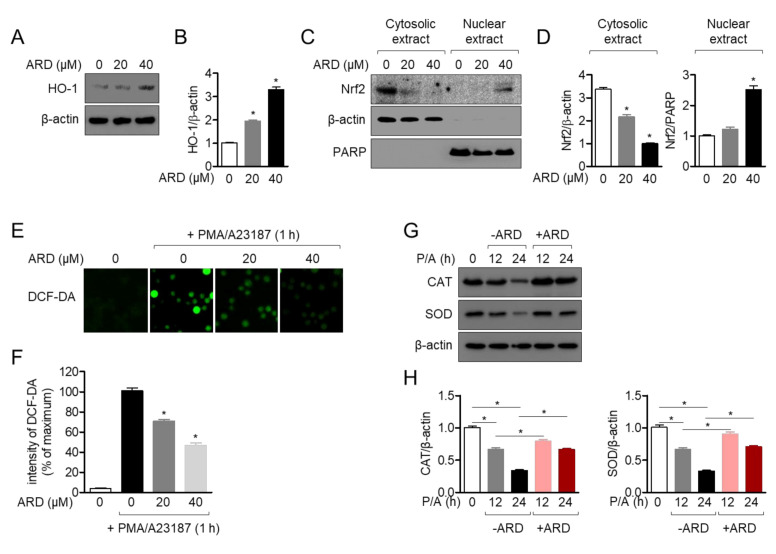
Aromadendrin induces the Nrf2 pathway and acts as an antioxidant in activated T cells. (**A**) The expression of HO-1 was detected by Western blot in Jurkat T cells treated with 20 or 40 μM of aromadendrin for 24 h. (**C**) The expression of Nrf2 in the cytosol and nucleus was determined by Western blot in Jurkat T cells treated with 20 or 40 μM of aromadendrin for 2 h. To separate nuclear extracts from whole lysate, a PE-NER kit was used. (**E**) Generated ROS (reactive oxygen species) was measured by 10 μM DCF-DA staining in Jurkat T cells pre-treated with 20 or 40 μM of aromadendrin for 24 h and treated with PMA (100 nM)/A23187 (1 μM) for 1 h. (**G**) Jurkat cells pre-treated with 40 μM aromadendrin for 24 h was stimulated with PMA (100 nM)/A23187 (1 μM) for 12 h or 24 h. After stimulation, cells were harvested, and the expression of catalase (CAT) and superoxide dismutase (SOD) was detected by Western blot. (**B**,**D**,**F**,**H**) The mean values ± SEM are presented in the bar graph. * *p* < 0.05, versus the control cells (**A**,**C**,**E**) or indicated two groups (G).

Aromadendrin has been reported that it has an antioxidative effect against 1, 1-diphenyl-2-picrylhydrazyl (DPPH) [14]. To evaluate whether the antioxidative effect of aromadendrin is mediated with immunosuppressive effect, we firstly checked the expression of heme oxygenase-1 (HO-1) after treatment with aromadendrin of Jurkat T cells for 24 h in dose-dependent manner. Figure 8A,B revealed that the expression of HO-1 was induced along with the concentration of aromadendrin. Since nuclear factor erythroid 2-related factor 2 (Nrf2) is highly responsible for HO-1 production, Nrf2 translocation into nucleus was also assessed by Western blot analysis. Figure 8C,D exhibited that Nrf2 is translocated into nucleus by treatment with aromadendrin in a dose-dependent manner. It has been reported that stimulation with PMA/A23187 induces an oxidative stress on T cells. To evaluate if enhanced HO-1 expression pre-treated with aromadendrin shows an antioxidative effect in activated T cells, the produced ROS (reactive oxygen species) was measured by staining 2′,7′-dichlorodihydrofluorescein diacetate (DCF-DA) reagent. Figure 8E,F showed that increased ROS production by PMA/A23187 stimulation was significantly reduced by pre-treatment with aromadendrin in Jurkat T cells. The expression of catalase (CAT) and superoxide dismutase (SOD) were also detected to confirm that aromadendrin reveals an antioxidative effect on PMA/A23187 stimulation by Western blot analysis. As shown in Figure 8G,H, the expressions of CAT and SOD were effectively restored by pre-treatment with aromadendrin. These results suggest that pre-treatment with aromadendrin enhances HO-1 expression and it effectively controls ROS production in activated T cells.

## 3. Discussion

The present study determined the regulatory effect of aromadendrin on T cell activation induced by anti-CD3 and anti-CD28 antibodies. Pre-treatment with aromadendrin suppressed IL-2 and IFNγ production and reduced the expression of functional surface molecules such as CD69, CD25, and CD40L from activated T cells. Aromadendrin blocked calcium influx in response to TCR-mediated stimulation and led to inhibition of NFAT translocation and activity in activated T cells. Pre-treatment with aromadendrin partially abrogated the NFκB and MAPK signaling pathways, without affecting the proximal signal pathway.

Calcium ions released from ER by stimulation of IP_3_ play a pivotal role for T cell activation. Calcium signaling possesses a variety of biological activities including induction of pro-inflammatory cytokines, differentiation into effector T cells, and expression of surface molecules on T cells [19]. CD69, CD25, and CD40L are well-defined molecules that act as functional surface molecules on receptors of cytokines or helper proteins to promote B cell differentiation [18]. The direct function of released calcium from ER to promote the expression of CD69 in T cells has been reported in the literature [20,21]. Our results from flow cytometry analysis demonstrated that TCR-mediated CD69, CD25, and CD40L expression levels were downregulated by pre-treatment with aromadendrin (Figure 4). The calcium influx in response to anti-CD3/CD28 antibodies was also effectively blocked by aromadendrin (Figure 5). These results suggest that aromadendrin intrinsically affects T cells activity, as well as extrinsically regulates the function of neighborhood immune cells including B cells.

Development of aromadendrin as a therapeutic agent for T cell-mediated diseases requires accurate and clear in vitro validation of target molecules of aromadendrin. Our results demonstrated that aromadendrin effectively controlled T cell activation through regulation of calcium influx and NFAT activity. Furthermore, aromadendrin exhibited a suppressive effect in calcium influx and significant reduction in translocation of NFAT as CsA, a calcium inhibitor, inhibited ( Figure 5; Figure 6); this may be indicative of a potential target of aromadendrin in calcium signaling being located in proximal signaling molecules of T cells or ER-related molecules, thus serving as calcium storage in cytosol. Our Western blot analysis showed contrasting results, which were contrary to aromadendrin’s involvement in proximal signal molecules (Figure 7); therefore, we assume that aromadendrin may physically bind to potential targets associated with ER such as calcium transporters, channels, and receptors [4]. A recent study on the inhibition of calcium influx into cytosol, via regulation of IP3 receptor in platelets by a polyphenol bioactive compound (epigallocatechin-3-gallate (EGCG)) derived from flavonoids [22], supports our findings on the suggested role of aromadendrin, as a modulator of T cell activation via regulation of calcium influx. Further studies should be carried out at the tissue level.

## 4. Materials and Methods

### 4.1. Cells

Jurkat T cells were purchased from the Korean Cell Line Bank (Seoul, Korea). Cells were cultured in RPMI (Rosewell Park Memorial Institute) medium (Welgene, Gyeongsan, Korea) supplemented with 10% fetal bovine serum (FBS), streptomycin (100 μg/mL), penicillin G (100 units/mL) and l-glutamine (2 mM). Cells were grown at 37 °C in a humidified incubator containing 5% CO_2_ and 95% air.

### 4.2. Reagents and Antibodies

AnnexinV and caspase3/7 staining reagents for IncuCyte^®^ cell imaging system was purchased from Essen bio (Ann Arbor, MI, USA). Antibodies against CD3 and CD28 for stimulation were obtained from Bioxcell (West Lebanon, NH, USA). Cyclosporine A (CsA), MTT (1-(4,5-Dimethylthiazol-2-yl)-3,5-diphenylformazan) powder, phorbol 12-myristate 13-acetate (PMA), A23187, Fluo 4, tacrolimus and DCF-DA were purchased from Sigma Chemical Co. (St. Louis, MO, USA). Staphylococcal enterotoxin E (SEE) was obtained from Toxin Technology (Sarasota, FL, USA). Human IL-2 and IFNγ DuoSet^®^ ELISA kit was purchased from R&D Systems (Minneapolis, MN, USA). ECL Western blotting detection reagents, NE-PER Nuclear and Cytoplasmic Extraction Reagents Kit and CFSE proliferation assay kit were obtained from Thermo Fisher Scientific (Waltham, MA, USA). Anti-CD69, anti-CD40L and anti-CD25 antibodies conjugated with APC were purchased from eBiosciences (San Diego, CA, USA). PVDF membrane was obtained from Bio-Rad (Hercules, CA, USA). Anti-NFAT, anti-β-actin, anti-HO-1, anti-Nrf2, anti-CAT and anti-SOD antibodies were purchased from Santa Cruz Biotechnology (Dallas, TX, USA). Anti-phosphorylated Lck (Y505), anti-phosphorylated ZAP70 (Y319), anti-ZAP70, anti-phosphorylated PLCγ1 (Y783), anti-phosphorylated PKCθ (T538), anti-PKCθ, anti-p65, anti-PARP, anti-IκBα, anti-phosphorylated IκBα (S32), anti-phosphorylated ERK (T202/Y204), anti-ERK, anti-phosphorylated p38 (T180/Y182), anti-p38, anti-phosphorylated JNK (T183/Y185), anti-JNK, anti-phosphorylated c-Jun (S73) and anti-c-Jun antibodies were obtained from Cell Signaling Technology (Danvers, MA, USA).

### 4.3. Isolation of Aromadendrin from C. Retusus Flowers

Aromadendrin (C_15_H_12_O_6_) was isolated from the flowers of *C. retusus*, as previously reported [11,23]. Briefly, the dried *C. retusus* flowers were extracted with MeOH, the MeOH extract was evaporated to under reduced pressure, and the residue was suspended in H_2_O. After that, MeOH extract was partitioned into EtOAc, n-BuOH and H_2_O. Among them, EtOAc fraction was separated to Sephadex LH-20 column chromatography under the elution condition of EtOAc-MeOH (15:15:1, 10:10:2) to obtain 10 fractions (Fr.1~10). Fraction 3 was separated to obtain five additional subfractions (Fr.3-1~5). Among them, subfraction Fr.3-2 was purified with Sephadex LH-20 (MeOH) to obtain Compound **1**. The isolated Compound **1** was identified as aromadendrin as a result of ^1^H and ^13^C-NMR (JEOL JNM-ECA 500) NMR spectroscopy analysis compared to published literature [11].

### 4.4. Determination of Confluency, Intensities of Annexinv and Caspase3/7 by IncuCyte

Jurkat T cells (5 × 10^3^/96-well plate) were incubated with the indicated concentration of aromadendrin (0 to 40 μM) for 24 h. Then, 1 μM of AnnexinV and caspase3/7 staining reagent were added to cells before incubation. Microscopic differential interference contrast (DIC) images were acquired by IncuCyte imaging system and cell confluency was assessed by IncuCyte Base software. Microscopic fluorescent images representing AnnexinV and caspase3/7 expressions were obtained by IncuCyte imaging system and integrated intensities were calculated by IncuCyte software. All intensities were normalized with intensity of unstimulated cells and shown in bar graph.

### 4.5. MTT Assay

Jurkat T cells (5 × 10^3^/96-well plate) were incubated with the indicated concentration of aromadendrin (0 to 40 μM) for 24 h. The supernatants were removed and 500 μg/mL of MTT were added to cells for 1 h. Supernatants were discarded, and generated formazan crystals were dissolved with 150 μL of DMSO. The plate was read to obtain the absorbance at 540 nm and cell viability was calculated by comparing with absorbance of control (% of control).

### 4.6. CFSE Proliferation Assay

Jurkat T cells were stained with 2 µM of CFSE for 30 min and washed with PBS. Cells were then treated with 0 to 40 µM aromadendrin for 24 h and acquired by flow cytometry. The percentage of proliferating cells were obtained by comparing CFSE peak of 0 h.

### 4.7. T Cell Stimulation

Jurkat T cells were pre-treated with aromadendrin at the indicated concentration (0 to 40 µM) or 40 µM for 1 h. Cells were placed on the plates coated with anti-CD3 (20 µg/mL) and soluble anti-CD28 (7 µg/mL) for the indicated time (3, 6 and 24 h). In some cases, PMA (100 nM) and A23187 (1 µM) were added to the pre-treated Jurkat T cells with aromadendrin. For superantigen stimulation, Raji B cells were pulsed with 1 µg/mL SEE for 30 min then Jurkat T cells were co-cultured with SEE-pulsed Raji B cells for the indicated time (3 and 6 h).

### 4.8. Conventional and Quantitative PCR for Measurement of mRNA Levels of Genes

Total RNA was isolated using TRIZOL reagent (JBI, Korea) and reverse transcription of the RNA to cDNA was performed using RT PreMix (Enzynomics, Daejeon, Korea). Primers used for each gene were as follows (forward and reverse primers, respectively): human *il2*, 5′-CAC GTC TTG CAC TTG TCA C-3′ and 5′-CCT TCT TGG GCA TGT AAA ACT-3′; human *ifng* 5′-TGG CTT TTC AGC TCT GCA TC-3′ and 5′-CCG CTA CAT CTG AAT GAC CTG-3′, human *gapdh*, 5′-CGG AGT CAA CGG ATT TGG TCG TAT-3′ and 5′-AGC CTT CTC CAT GGT GGT GAA GAC-3′. The condition of conventional PCR was as follows: 28 cycles of denaturation at 95 °C for 30 s, annealing at 60 °C for 20 s, and extension at 72 °C for 40 s; followed by denaturation at 72 °C for 7 min. For quantitative PCR analysis, PCR amplification was performed in a DNA Engine Opticon 1 continuous fluorescence detection system (MJ Research, Waltham, MA, USA) by using SYBR Premix Ex Taq (Takara, Japan). The total reaction volume was 10 μL containing 0.1 μg of cDNA. Each PCR reaction was performed using the following conditions: 95 °C for 30 s, 60 °C for 30 s, and plate read (detection of fluorescent product) for 40 cycles followed by 7 min of extension at 72 °C. Melting curve analysis was performed to characterize the dsDNA product by slowly raising the temperature (0.2 °C/s) from 60 °C to 95 °C with fluorescence data collected at 0.2 °C intervals. mRNA levels of *il2* and *ifng* were normalized with mRNA levels of *gapdh* and were presented as % of controls. The fold change in gene expression was calculated using the following equation: fold change = 2 − ΔΔCT, where ΔΔCT = (CTtarget − CTgapdh) at time x − (CTtarget − CTgapdh) at time 0. Here, time x represents any time point and time 0 represents the 1 X expression of the target gene in the control cells normalized to *gapdh*.

### 4.9. IL-2 and IFNγ Measurement by ELISA

Jurkat T cells (5 × 10^3^/well, 96-well plate) were stimulated with immobilized anti-CD3 antibodies (20 μg/mL) and soluble anti-CD28 (7 μg/mL) antibodies for 24 h. After incubation, supernatants were collected from activated T cells and the amounts of produced IL-2 and IFNγ were determined by a DuoSet^®^ ELISA kit (R&D Systems, Minneapolis, MN, USA) following manufacture’s instruction.

### 4.10. Assessment of Surface Molecules Expression by Flow Cytometry

The expressions of CD69, CD40L and CD25 on the T cell surface were measured by flow cytometry. Placed Jurkat T cells on the plate coated with anti-CD3 and incubated with soluble anti-CD28 antibodies for the 16 h (CD69) or 24 h (CD40L and CD25) were harvested and stained with antibodies conjugated with APC at 4 °C for 30 min. CD69, CD40L and CD25 expressions on the T cell surface were acquired by flow cytometry and the expressions were presented in a histogram graph. Mean fluorescence intensities were shown in a bar graph.

### 4.11. Calcium Measurement

Jurkat T cells (1 × 10^5^/mL) were stained with 4 μM of Fluo-4 following manufacture’s instruction. After pre-treatment of the stained Jurkat T cells with 40 μM of ARD or 1 μM of CsA for 1 h, cells were stimulated by soluble anti-CD3 (20 μg/mL) and anti-CD28 (7 μg/mL) and fluorescence was acquired by flow cytometry, IncuCyte and an immunoabsorbance reader.

### 4.12. Western Blot

Stimulated Jurkat T cells in the indicated conditions were collected for lysis in RIPA buffer with phosphatase inhibitor at 4 °C for 30 min. For separation of nucleic extracts from whole lysate, an NE-PER Nuclear and Cytoplasmic Extraction Reagents Kit was used following manufacture’s instruction. Lysates were centrifuged at 14,000 rpm at 4 °C for 30 min, and approximately 40 μg of the lysate was separated on 8–12% SDS-PAGE gels. Proteins were transferred on PVDF membranes. Membranes were blocked in 5% skim milk for 1 h and incubated with indicated primary antibodies in 3% skim milk overnight. Excess primary antibodies were discarded by washing the membrane five times with TBS-T and incubated with 0.1 μg/mL peroxidase-labeled secondary antibodies (against rabbit or mouse) for 2 h. After five washes with TBS-T, bands were visualized with ECL Western blotting detection reagents with an ImageQuant LAS 4000 (GE healthcare, Chicago, IL, USA).

### 4.13. ROS Measurement

Jurkat cells were pre-treated with 20 or 40 µM of aromadendrin for 24 h and stimulated with PMA (100 nM)/A23187 (1 µM) for 1 h. After stimulation, cells were stained with 10 µM DCF-DA for 30 min and generated fluorescence was acquired by IncuCyte imaging system. The intensity of DCF-DA was automatically calculated in IncuCyte base software.

### 4.14. Statistics

Mean values ± SEM (standard error of the mean) were calculated from the data acquired from three independent experiments performed on separate days and presented in graph. One-way ANOVA was used to obtain significance (*p* value). * indicates differences between indicated groups considered significant at *p* < 0.05.
